# Dibromido(2,3-di-2-pyridyl­pyrazine-κ^2^ *N* ^1^,*N* ^2^)platinum(II)

**DOI:** 10.1107/S1600536811031643

**Published:** 2011-08-11

**Authors:** Kwang Ha

**Affiliations:** a School of Applied Chemical Engineering, The Research Institute of Catalysis, Chonnam National University, Gwangju 500-757, Republic of Korea

## Abstract

The Pt^II^ ion in the title complex, [PtBr_2_(C_14_H_10_N_4_)], is four-coordinated in a distorted square-planar environment by two N atoms of a chelating 2,3-di-2-pyridyl­pyrazine ligand and two bromide anions. In the crystal, the pyridyl ring coordinated to the Pt atom is inclined slightly to its carrier pyrazine ring [dihedral angle = 14.7 (2)°], whereas the uncoordinated pyridyl ring is inclined considerably to the pyrazine ring [dihedral angle = 51.9 (3)°]. The dihedral angle between the two pyridyl rings is 57.7 (3)°. Two complex mol­ecules are assembled through inter­molecular C—H⋯N hydrogen bonds, forming a dimer-type species. Intra­molecular C—H⋯Br and C—H⋯N hydrogen bonds are also present.

## Related literature

For the crystal structure of [PtCl_4_(dpp)] (dpp is 2,3-di-2-pyridyl­pyrazine), see: Delir Kheirollahi Nezhad *et al.* (2008 ▶).
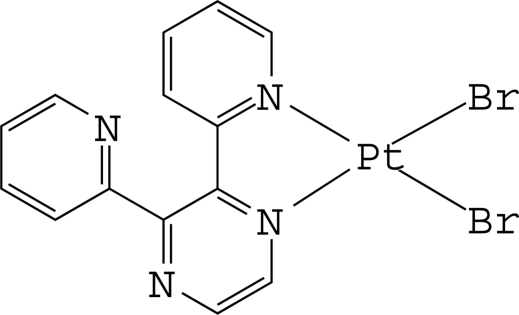


## Experimental

### 

#### Crystal data

[PtBr_2_(C_14_H_10_N_4_)]
*M* *_r_* = 589.14
Monoclinic, 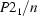

*a* = 8.9084 (11) Å
*b* = 9.9817 (12) Å
*c* = 16.727 (2) Å
β = 94.104 (3)°
*V* = 1483.6 (3) Å^3^
*Z* = 4
Mo *K*α radiation
μ = 14.84 mm^−1^
*T* = 200 K
0.17 × 0.10 × 0.04 mm

#### Data collection

Bruker SMART 1000 CCD diffractometer
Absorption correction: multi-scan (*SADABS*; Bruker, 2001 ▶) *T*_min_ = 0.590, *T*_max_ = 1.000
10590 measured reflections
3642 independent reflections
2415 reflections with *I* > 2σ(*I*)
*R*_int_ = 0.060

#### Refinement

*R*[*F* ^2^ > 2σ(*F* ^2^)] = 0.040
*wR*(*F* ^2^) = 0.099
*S* = 1.01
3642 reflections
190 parameters
H-atom parameters constrained
Δρ_max_ = 3.12 e Å^−3^
Δρ_min_ = −1.54 e Å^−3^

### 

Data collection: *SMART* (Bruker, 2007 ▶); cell refinement: *SAINT* (Bruker, 2007 ▶); data reduction: *SAINT*; program(s) used to solve structure: *SHELXS97* (Sheldrick, 2008 ▶); program(s) used to refine structure: *SHELXL97* (Sheldrick, 2008 ▶); molecular graphics: *ORTEP-3* (Farrugia, 1997 ▶) and *PLATON* (Spek, 2009 ▶); software used to prepare material for publication: *SHELXL97*.

## Supplementary Material

Crystal structure: contains datablock(s) global, I. DOI: 10.1107/S1600536811031643/hy2455sup1.cif

Structure factors: contains datablock(s) I. DOI: 10.1107/S1600536811031643/hy2455Isup2.hkl

Additional supplementary materials:  crystallographic information; 3D view; checkCIF report

## Figures and Tables

**Table 1:** Selected bond lengths (Å)

Pt1—N1	2.020 (6)
Pt1—N3	2.033 (8)
Pt1—Br1	2.4116 (11)
Pt1—Br2	2.4142 (10)

**Table 2:** Hydrogen-bond geometry (Å, °)

*D*—H⋯*A*	*D*—H	H⋯*A*	*D*⋯*A*	*D*—H⋯*A*
C3—H3⋯N2^i^	0.95	2.55	3.396 (11)	148
C4—H4⋯Br1	0.95	2.66	3.289 (9)	124
C6—H6⋯N4	0.95	2.59	3.051 (11)	110
C9—H9⋯Br2	0.95	2.71	3.340 (10)	124

Symmetry code: (i) 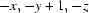.

**Fig. 1.**
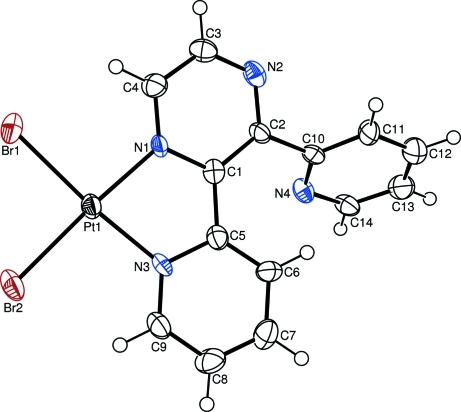
The molecular structure of the title complex, with displacement ellipsoids drawn at the 50% probability level.

**Fig. 2.**
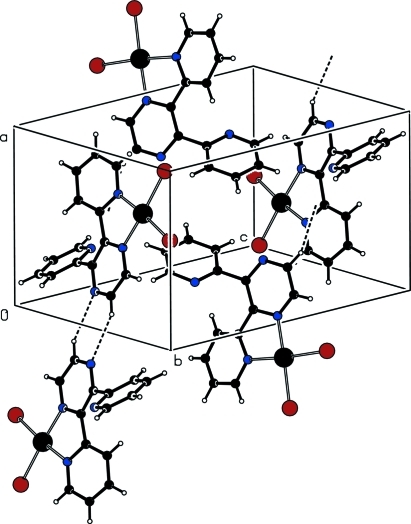
View of crystal packing of the title complex. Intermolecular hydrogen bonds are drawn as dashed lines.

## supplementary crystallographic information

### Comment

In the title complex, [PtBr_2_(dpp)] (dpp is 2,3-di-2-pyridylpyrazine, C_14_H_10_N_4_), the Pt^II^ ion is four-coordinated in a distorted square-planar environment by two N atoms from the pyrazine ring and one pyridyl ring of the chelating dpp ligand and two bromide anions (Fig. 1). The coordination mode of the dpp ligand is similar to that of a mononuclear Pt(IV) complex [PtCl_4_(dpp)] (Delir Kheirollahi Nezhad *et al.*, 2008).

The main contribution to the distortion of the square-plane is the tight N1—Pt1—N3 chelate angle of 80.4 (3)°, which results in slightly bent *trans* axes [Br1—Pt1—N3 = 175.09 (18) and Br2—Pt1—N1 = 176.6 (2)°]. The Pt—N and Pt—Br bond lengths are nearly equivalent, respectively (Table 1). In the crystal, the pyridyl ring coordinated to the Pt atom is located slightly inclined to its carrier pyrazine ring, making a dihedral angle of 14.7 (2)°. On the contrary, the uncoordinated pyridyl ring is considerably inclined to the pyrazine ring with a dihedral angle of 51.9 (3)°. The dihedral angle between the two pyridyl rings is 57.7 (3)°. Two complex molecules are assembled through intermolecular C—H···N hydrogen bonds, forming a dimer-type species (Fig. 2 and Table 2). There are also intramolecular C—H···N and C—H···Br hydrogen bonds (Table 2). The complexes stack in columns along the *c* axis.

### Experimental

To a solution of K_2_PtBr_4_ (0.297 g, 0.500 mmol) in H_2_O (20 ml) was added 2,3-di-2-pyridylpyrazine (0.117 g, 0.501 mmol) and stirred for 3 h at room temperature. The formed precipitate was separated by filtration, washed with H_2_O and acetone and dried at 50 °C, to give a redbrown powder (0.133 g). Crystals suitable for X-ray analysis were obtained by slow evaporation from an acetone solution.

### Refinement

H atoms were positioned geometrically and allowed to ride on their respective parent atoms [C—H = 0.95 Å and *U*_iso_(H) = 1.2*U*_eq_(C)]. The highest peak (3.12 e Å^-3^) and the deepest hole (-1.54 e Å^-3^) in the difference Fourier map are located 0.97 Å and 0.94 Å from the atoms Br1 and Pt1, respectively.

### Crystal data

**Table:**

[PtBr_2_(C_14_H_10_N_4_)]	*F*(000) = 1080
*M**_r_* = 589.14	*D*_x_ = 2.638 Mg m^−^^3^
Monoclinic, *P*2_1_/*n*	Mo *K*α radiation, λ = 0.71073 Å
Hall symbol: -P 2yn	Cell parameters from 3431 reflections
*a* = 8.9084 (11) Å	θ = 2.4–27.9°
*b* = 9.9817 (12) Å	µ = 14.84 mm^−^^1^
*c* = 16.727 (2) Å	*T* = 200 K
β = 94.104 (3)°	Needle, orange
*V* = 1483.6 (3) Å^3^	0.17 × 0.10 × 0.04 mm
*Z* = 4	

### Data collection

**Table:**

Bruker SMART 1000 CCD diffractometer	3642 independent reflections
Radiation source: fine-focus sealed tube	2415 reflections with *I* > 2σ(*I*)
graphite	*R*_int_ = 0.060
φ and ω scans	θ_max_ = 28.3°, θ_min_ = 2.4°
Absorption correction: multi-scan (*SADABS*; Bruker, 2001)	*h* = −11→11
*T*_min_ = 0.590, *T*_max_ = 1.000	*k* = −8→13
10590 measured reflections	*l* = −22→21

### Refinement

**Table:**

Refinement on *F*^2^	Primary atom site location: structure-invariant direct methods
Least-squares matrix: full	Secondary atom site location: difference Fourier map
*R*[*F*^2^ > 2σ(*F*^2^)] = 0.040	Hydrogen site location: inferred from neighbouring sites
*wR*(*F*^2^) = 0.099	H-atom parameters constrained
*S* = 1.01	*w* = 1/[σ^2^(*F*_o_^2^) + (0.0359*P*)^2^] where *P* = (*F*_o_^2^ + 2*F*_c_^2^)/3
3642 reflections	(Δ/σ)_max_ = 0.001
190 parameters	Δρ_max_ = 3.12 e Å^−^^3^
0 restraints	Δρ_min_ = −1.54 e Å^−^^3^

### Fractional atomic coordinates and isotropic or equivalent isotropic displacement parameters (Å^2^)

**Table:**

	*x*	*y*	*z*	*U*_iso_*/*U*_eq_	
Pt1	0.63258 (4)	0.64063 (4)	0.11956 (2)	0.02665 (12)	
Br1	0.54983 (11)	0.86499 (10)	0.08593 (7)	0.0451 (3)	
Br2	0.88077 (10)	0.72974 (11)	0.15395 (6)	0.0399 (3)	
N1	0.4307 (7)	0.5552 (7)	0.0900 (4)	0.0237 (16)	
N2	0.1622 (8)	0.4208 (8)	0.0613 (4)	0.0310 (18)	
N3	0.6852 (7)	0.4458 (7)	0.1435 (4)	0.0248 (16)	
N4	0.2993 (8)	0.1889 (8)	0.1962 (4)	0.0299 (17)	
C1	0.4250 (9)	0.4200 (9)	0.1031 (4)	0.0235 (19)	
C2	0.2830 (9)	0.3560 (9)	0.0921 (5)	0.0256 (19)	
C3	0.1765 (9)	0.5492 (10)	0.0439 (5)	0.031 (2)	
H3	0.0934	0.5948	0.0176	0.038*	
C4	0.3059 (9)	0.6182 (10)	0.0624 (5)	0.032 (2)	
H4	0.3073	0.7127	0.0555	0.038*	
C5	0.5715 (10)	0.3561 (9)	0.1244 (5)	0.028 (2)	
C6	0.6014 (9)	0.2217 (10)	0.1232 (5)	0.033 (2)	
H6	0.5237	0.1600	0.1073	0.039*	
C7	0.7453 (11)	0.1756 (10)	0.1454 (5)	0.039 (2)	
H7	0.7677	0.0827	0.1439	0.047*	
C8	0.8532 (11)	0.2654 (10)	0.1693 (6)	0.040 (2)	
H8	0.9505	0.2352	0.1881	0.049*	
C9	0.8223 (10)	0.4001 (10)	0.1664 (6)	0.036 (2)	
H9	0.9004	0.4622	0.1810	0.044*	
C10	0.2543 (8)	0.2169 (9)	0.1196 (5)	0.0259 (19)	
C11	0.1743 (10)	0.1274 (9)	0.0696 (5)	0.033 (2)	
H11	0.1431	0.1509	0.0159	0.040*	
C12	0.1409 (11)	0.0019 (11)	0.1004 (6)	0.043 (3)	
H12	0.0878	−0.0631	0.0680	0.052*	
C13	0.1867 (10)	−0.0253 (10)	0.1786 (6)	0.040 (2)	
H13	0.1644	−0.1094	0.2016	0.048*	
C14	0.2650 (9)	0.0699 (10)	0.2235 (5)	0.032 (2)	
H14	0.2964	0.0487	0.2775	0.039*	

### Atomic displacement parameters (Å^2^)

**Table:**

	*U*^11^	*U*^22^	*U*^33^	*U*^12^	*U*^13^	*U*^23^
Pt1	0.02580 (19)	0.0228 (2)	0.03144 (19)	−0.00629 (16)	0.00245 (13)	−0.00065 (17)
Br1	0.0445 (6)	0.0213 (5)	0.0690 (7)	−0.0049 (5)	0.0003 (5)	0.0012 (5)
Br2	0.0336 (5)	0.0397 (6)	0.0459 (5)	−0.0179 (5)	−0.0002 (4)	0.0005 (5)
N1	0.024 (4)	0.018 (4)	0.029 (4)	−0.007 (3)	−0.004 (3)	0.001 (3)
N2	0.024 (4)	0.036 (5)	0.034 (4)	−0.009 (4)	0.005 (3)	−0.004 (4)
N3	0.019 (3)	0.029 (4)	0.026 (3)	−0.008 (3)	−0.003 (3)	−0.002 (3)
N4	0.027 (4)	0.032 (5)	0.032 (4)	−0.009 (3)	0.003 (3)	0.000 (4)
C1	0.029 (5)	0.025 (5)	0.017 (4)	−0.004 (4)	0.005 (3)	0.001 (4)
C2	0.016 (4)	0.028 (5)	0.033 (4)	−0.003 (4)	0.001 (3)	−0.004 (4)
C3	0.022 (4)	0.040 (6)	0.033 (5)	0.001 (4)	0.005 (4)	0.002 (5)
C4	0.030 (5)	0.031 (6)	0.034 (5)	−0.001 (4)	0.001 (4)	0.009 (4)
C5	0.031 (5)	0.026 (5)	0.026 (4)	−0.004 (4)	−0.001 (3)	−0.001 (4)
C6	0.023 (5)	0.032 (6)	0.043 (5)	0.005 (4)	0.000 (4)	0.003 (5)
C7	0.045 (6)	0.026 (6)	0.047 (6)	−0.001 (5)	0.011 (5)	0.001 (5)
C8	0.038 (5)	0.036 (6)	0.047 (6)	0.006 (5)	0.001 (4)	0.016 (5)
C9	0.026 (5)	0.036 (6)	0.047 (5)	−0.009 (4)	0.001 (4)	−0.005 (5)
C10	0.014 (4)	0.030 (5)	0.034 (5)	−0.002 (4)	0.002 (3)	0.000 (4)
C11	0.039 (5)	0.022 (5)	0.038 (5)	−0.007 (4)	−0.005 (4)	0.007 (5)
C12	0.044 (6)	0.033 (6)	0.050 (6)	−0.004 (5)	−0.008 (5)	0.003 (5)
C13	0.033 (5)	0.034 (6)	0.054 (6)	−0.003 (5)	0.004 (5)	0.011 (5)
C14	0.024 (5)	0.038 (6)	0.033 (5)	−0.006 (4)	−0.003 (4)	0.010 (5)

### Geometric parameters (Å, °)

**Table:**

Pt1—N1	2.020 (6)	C4—H4	0.9500
Pt1—N3	2.033 (8)	C5—C6	1.368 (12)
Pt1—Br1	2.4116 (11)	C6—C7	1.387 (12)
Pt1—Br2	2.4142 (10)	C6—H6	0.9500
N1—C4	1.330 (10)	C7—C8	1.353 (13)
N1—C1	1.369 (11)	C7—H7	0.9500
N2—C3	1.323 (11)	C8—C9	1.373 (13)
N2—C2	1.327 (10)	C8—H8	0.9500
N3—C9	1.334 (11)	C9—H9	0.9500
N3—C5	1.372 (11)	C10—C11	1.386 (12)
N4—C14	1.317 (11)	C11—C12	1.396 (13)
N4—C10	1.344 (10)	C11—H11	0.9500
C1—C2	1.417 (11)	C12—C13	1.369 (13)
C1—C5	1.474 (12)	C12—H12	0.9500
C2—C10	1.491 (12)	C13—C14	1.370 (13)
C3—C4	1.360 (12)	C13—H13	0.9500
C3—H3	0.9500	C14—H14	0.9500
			
N1—Pt1—N3	80.4 (3)	N3—C5—C1	113.6 (8)
N1—Pt1—Br1	94.7 (2)	C5—C6—C7	119.9 (9)
N3—Pt1—Br1	175.09 (18)	C5—C6—H6	120.1
N1—Pt1—Br2	176.6 (2)	C7—C6—H6	120.1
N3—Pt1—Br2	96.39 (18)	C8—C7—C6	118.9 (9)
Br1—Pt1—Br2	88.44 (4)	C8—C7—H7	120.6
C4—N1—C1	118.8 (7)	C6—C7—H7	120.6
C4—N1—Pt1	126.3 (6)	C7—C8—C9	120.1 (9)
C1—N1—Pt1	114.8 (5)	C7—C8—H8	119.9
C3—N2—C2	118.0 (7)	C9—C8—H8	119.9
C9—N3—C5	119.3 (8)	N3—C9—C8	121.5 (9)
C9—N3—Pt1	125.2 (6)	N3—C9—H9	119.3
C5—N3—Pt1	115.0 (6)	C8—C9—H9	119.3
C14—N4—C10	117.2 (8)	N4—C10—C11	123.2 (8)
N1—C1—C2	117.8 (8)	N4—C10—C2	116.2 (8)
N1—C1—C5	115.0 (7)	C11—C10—C2	120.4 (8)
C2—C1—C5	127.1 (8)	C10—C11—C12	118.0 (8)
N2—C2—C1	121.5 (8)	C10—C11—H11	121.0
N2—C2—C10	114.9 (7)	C12—C11—H11	121.0
C1—C2—C10	123.4 (7)	C13—C12—C11	118.3 (9)
N2—C3—C4	122.3 (8)	C13—C12—H12	120.8
N2—C3—H3	118.9	C11—C12—H12	120.8
C4—C3—H3	118.9	C12—C13—C14	119.4 (9)
N1—C4—C3	120.9 (9)	C12—C13—H13	120.3
N1—C4—H4	119.6	C14—C13—H13	120.3
C3—C4—H4	119.6	N4—C14—C13	123.9 (8)
C6—C5—N3	120.2 (8)	N4—C14—H14	118.1
C6—C5—C1	126.2 (8)	C13—C14—H14	118.1
			
N3—Pt1—N1—C4	−179.5 (7)	Pt1—N3—C5—C1	−9.9 (9)
Br1—Pt1—N1—C4	−0.3 (7)	N1—C1—C5—C6	−164.7 (8)
N3—Pt1—N1—C1	2.9 (5)	C2—C1—C5—C6	12.9 (14)
Br1—Pt1—N1—C1	−177.8 (5)	N1—C1—C5—N3	12.5 (10)
N1—Pt1—N3—C9	175.8 (7)	C2—C1—C5—N3	−169.9 (7)
Br2—Pt1—N3—C9	−3.0 (7)	N3—C5—C6—C7	3.3 (13)
N1—Pt1—N3—C5	4.1 (6)	C1—C5—C6—C7	−179.6 (8)
Br2—Pt1—N3—C5	−174.7 (5)	C5—C6—C7—C8	1.2 (13)
C4—N1—C1—C2	−4.7 (11)	C6—C7—C8—C9	−4.2 (14)
Pt1—N1—C1—C2	173.1 (5)	C5—N3—C9—C8	1.6 (13)
C4—N1—C1—C5	173.1 (7)	Pt1—N3—C9—C8	−169.7 (7)
Pt1—N1—C1—C5	−9.1 (9)	C7—C8—C9—N3	2.9 (14)
C3—N2—C2—C1	−2.7 (12)	C14—N4—C10—C11	0.3 (12)
C3—N2—C2—C10	172.7 (7)	C14—N4—C10—C2	175.0 (7)
N1—C1—C2—N2	7.8 (12)	N2—C2—C10—N4	−124.7 (8)
C5—C1—C2—N2	−169.7 (7)	C1—C2—C10—N4	50.6 (11)
N1—C1—C2—C10	−167.3 (7)	N2—C2—C10—C11	50.1 (11)
C5—C1—C2—C10	15.2 (13)	C1—C2—C10—C11	−134.5 (9)
C2—N2—C3—C4	−5.4 (13)	N4—C10—C11—C12	−0.8 (13)
C1—N1—C4—C3	−3.0 (12)	C2—C10—C11—C12	−175.3 (8)
Pt1—N1—C4—C3	179.5 (6)	C10—C11—C12—C13	1.1 (14)
N2—C3—C4—N1	8.5 (13)	C11—C12—C13—C14	−0.9 (14)
C9—N3—C5—C6	−4.7 (12)	C10—N4—C14—C13	−0.2 (13)
Pt1—N3—C5—C6	167.5 (6)	C12—C13—C14—N4	0.5 (15)
C9—N3—C5—C1	177.9 (7)		

### Hydrogen-bond geometry (Å, °)

**Table:**

*D*—H···*A*	*D*—H	H···*A*	*D*···*A*	*D*—H···*A*
C3—H3···N2^i^	0.95	2.55	3.396 (11)	148.
C4—H4···Br1	0.95	2.66	3.289 (9)	124.
C6—H6···N4	0.95	2.59	3.051 (11)	110.
C9—H9···Br2	0.95	2.71	3.340 (10)	124.

Symmetry codes: (i) −*x*, −*y*+1, −*z*.
